# Supplementary material for: Speech emotion recognition using machine learning techniques: Feature extraction and comparison of convolutional neural network and random forest
Source: PLoS One. 2023 Nov 21;18(11):e0291500. doi: 10.1371/journal.pone.0291500 (PMC10662716; doi:10.1371/journal.pone.0291500)
Supplement: Attachment — Submitted filename: Reviewer.docx [file pone.0291500.s001.docx]

Reviewer #1: The authors have presented a machine learning based speech emotion classification method. There are few concerns on the work:

1) Emo-DB is an important dataset, which the authors have missed in their work.

*We appreciate your suggestion to include the Emo-DB dataset in our work. However, we did not use the Emo-DB dataset due to various reasons, one of which is that it is in the German language. Instead, we used other datasets such as RAVDESS, CREMA, TESS, and SAVEE, which are in English. In addition, we combine all those datasets, instead of evaluating them individually. We hope this clarifies our choice of datasets for our work.*

2) The literature review is not adequate.

*We have expanded the literature review in our work based on your comments to include more relevant sources and provide a more comprehensive overview of the topic.*

3) The results are not comparable to the state-of-the-art methods on the same dataset.

For a details of the literature and comparison, please refer to this article: https://arxiv.org/pdf/2112.05666.pdf or similar articles

*That was a great point and added in our work. It should be noted that the majority of those studies considered a single dataset, one at a time. We added the below point to our work”*

*The majority of past studies that evaluated audio for emotion recognition, considered audio dataset individually which might be biased due to invariability of the dataset. For instance, an ensemble 1D-CNN-LSTM-GRU was used [10], where the model achieved a promising results while considering a single dataset at a time, For instance, the model achieved 95.42% for TESS, 95.62 for RAVDESS, and 93.22% for SAVEE datasets. Or for instance, [11] applied their hybrid CNN- LSTM models on 6 different datasets separately. They achieved, for instance, 90.42% accuracy for RADVDESS, or 99.48% for the TESS datasets. In contrast, our approach combines multiple datasets to provide a more comprehensive evaluation of audio emotion recognition. “*

*Finally, it should be noted that we added the comments in the data section to address the reviewer’s concern regarding the availability of the employed datasets. “*

*It is important to note that all of the datasets used in our study are publicly available and can be accessed through the references provided for each dataset.*

*“*

*Thank you for your comments. We appreciate the time and effort that the reviewer has taken to provide feedback on our work. If you have any further concerns or suggestions, please don’t hesitate to let us know. We value your input and are always looking for ways to improve our work.*

Reviewer #2: 1. Please use single space tab for every para.
2. Represent abbreviations RAVDESS, TESS etc in caps in your paper.

*Those are double checked to make sure, they are consistent,*
3. Spectrographic representation of speech emotions are missing, represent them.

Sure, those are presented in Figure 1 as follows:”

Various characteristics of audios were presented in Figure 1.In the left figure, the figure computed short-time Fourier transform (STFT) of the audio data. The right figure present short-time Fourier transform (STFT) of the audio data. The STFT is then converted to decibels. It could be seen that the differences between emotions such as anger and sadness are not easily distinguishable in the waveplot and spectrogram figures.

Emotions are complex and can be expressed in many different ways, and it can be challenging to accurately classify emotions based on audio data alone. That is evident from the visual representations of audio data, such as waveplots and spectrograms, which may not always clearly show the differences between emotions.

|  |  |
| --- | --- |
|  |  |

Figure 1. Audio waveplot and spectrogram of fear and anger emotions.

4. Graphical representation of results also missing as a comparision.

We considered the below figure “

*We have included Figure 3 to provide a visual representation of the distribution of accuracy for various emotions. As can be seen from Figure 3, surprise has the best performance, while disgust has the lowest performance..*

*Figure 3. Comparison of CONV1D and Random Forest models for audio emotion classification.*

*Thank you for your comments. We appreciate the time and effort that the reviewer has taken to provide feedback on our work. If you have any further concerns or suggestions, please don’t hesitate to let us know. We value your input and are always looking for ways to improve our work.*
